# Non-random walk diffusion enhances the sink strength of semicoherent interfaces

**DOI:** 10.1038/ncomms10424

**Published:** 2016-01-29

**Authors:** A. Vattré, T. Jourdan, H. Ding, M.-C. Marinica, M. J. Demkowicz

**Affiliations:** 1 CEA, DAM, DIF, F-91297 Arpajon, France; 2 CEA, DEN, Service de Recherches de Métallurgie Physique, Université Paris-Saclay, F-91191 Gif-sur-Yvette, France; 3 Department of Materials Science and Engineering, Massachusetts Institute of Technology, Cambridge, Massachusetts 02139, USA; 4 Materials Science and Engineering, Texas A&M University, College Station, Texas 77843, USA

## Abstract

Clean, safe and economical nuclear energy requires new materials capable of withstanding severe radiation damage. One strategy of imparting radiation resistance to solids is to incorporate into them a high density of solid-phase interfaces capable of absorbing and annihilating radiation-induced defects. Here we show that elastic interactions between point defects and semicoherent interfaces lead to a marked enhancement in interface sink strength. Our conclusions stem from simulations that integrate first principles, object kinetic Monte Carlo and anisotropic elasticity calculations. Surprisingly, the enhancement in sink strength is not due primarily to increased thermodynamic driving forces, but rather to reduced defect migration barriers, which induce a preferential drift of defects towards interfaces. The sink strength enhancement is highly sensitive to the detailed character of interfacial stresses, suggesting that ‘super-sink' interfaces may be designed by optimizing interface stress fields. Such interfaces may be used to create materials with unprecedented resistance to radiation-induced damage.

## Abstract (web summary)

Materials resistant to radiation damage could improve the safety, efficiency and sustainability of nuclear energy. Here, the authors suggest that some materials and interfaces may be effective sinks for defects caused by this damage and propose that elastic interactions enhance sink strength.

## Graphical abstract

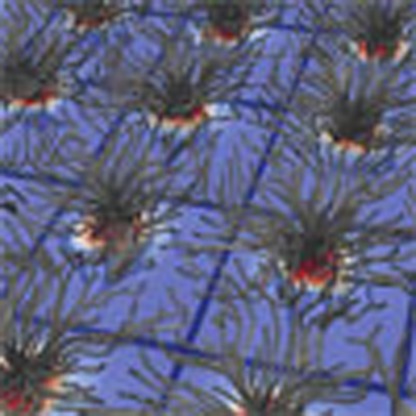

Damage caused by high-energy particles, such as neutrons or ions, severely limits the performance of materials used in nuclear energy applications^1^,^2^. It arises from the exceedingly high concentrations—many orders of magnitude greater than under thermodynamic equilibrium—of crystal defects created by impinging radiation^3^,^4^. Preventing radiation-induced damage in engineering solids requires rapid removal of these defects. Materials resistant to radiation damage would markedly improve the safety, efficiency and sustainability of nuclear energy.

One way of removing radiation-induced defects is to provide a high density of sinks, such as grain boundaries or heterophase interfaces^5^,^6^,^7^ that continually absorb defects as they are created. This motivation underlies ongoing exploration of the radiation response of nanocrystalline and nanocomposite materials^8^,^9^,^10^,^11^, because of the large total interface area per unit volume they contain. These investigations have demonstrated wide variations in sink behaviour of different interfaces. Some easily absorb defects, preventing damage in neighbouring material, but become damaged themselves^12^. Others are poor sinks for isolated defects, but excellent sinks for defect clusters^13^,^14^. The sink behaviour of yet others changes with radiation dose^15^,^16^,^17^. This wide variety of radiation responses prompts us to ask: are some specific interfaces best suited to mitigate radiation damage? Is it possible to identify them without resorting to resource-intensive irradiation experiments?

To answer these questions, we propose an improved computational method for rapidly assessing the vacancy and interstitial sink strength of semicoherent interfaces. This method builds on a reduced order model for elastic fields of heterophase bicrystals^18^. Such interfaces are of particular interest because many of them contain a high density of defect trapping sites^19^,^20^,^21^. Moreover, semicoherent interfaces generate elastic fields that interact directly with radiation-induced defects^18^,^22^,^23^. We show that these elastic fields have an unexpectedly large influence on interface sink strength. Unlike previous studies, which highlighted the importance of thermodynamic driving forces for interface sink behaviour^24^,^25^,^26^, we find that the principal effect of the elastic fields is to modify defect diffusivities, causing defects to drift preferentially towards the interface through a non-random walk process. Our work also demonstrates that interface sink strength is highly sensitive to the exact distribution of interface elastic fields. These findings motivate a computational search for ‘super-sink' interfaces: ones that optimally attract, absorb and annihilate radiation-induced defects.

## Results

### Kinetic Monte Carlo simulations with elastic interactions

Modelling the removal of radiation-induced point defects at sinks is a challenging task: on one hand, the variety and complexity of defect behaviours call for the flexibility of atomistic modelling. On the other, the relatively slow, thermally activated mechanisms of defect motion require longer simulation times than may be reached using conventional atomistic techniques, such as molecular dynamics. We employ the object kinetic Monte Carlo (OKMC) method^27^,^28^,^29^,^30^, which is well suited to modelling long-time, thermally activated processes yet is also able to account for nuances of defect behaviour uncovered through atomistic modelling.

**Figure 1:**
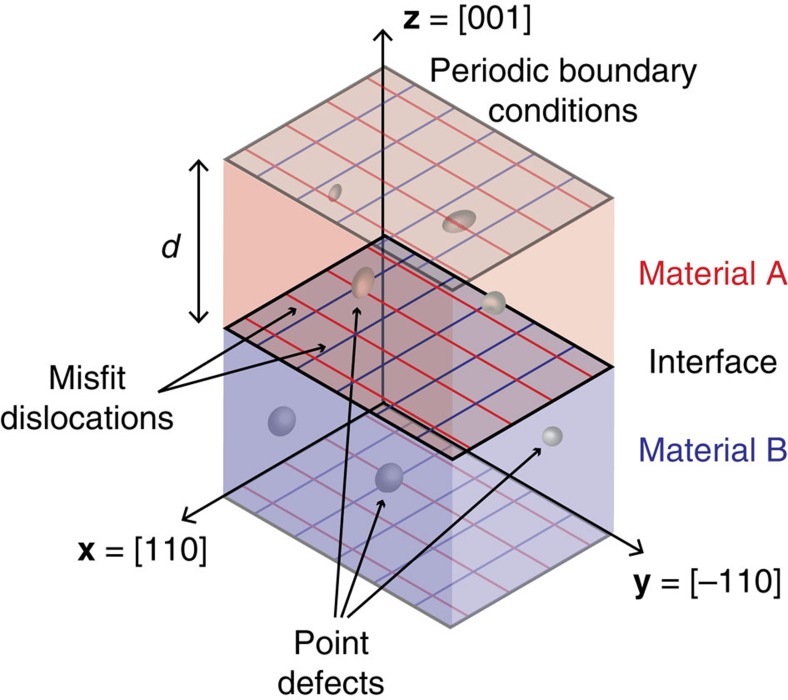
Schematic illustration of our model. We simulate the diffusion of radiation-induced point defects (illustrated by ovals) to interfaces under the influence of interface elastic fields. In general, materials A and B may be any two crystalline solids. In the present work, they are chosen to be either Cu or Ag (see Table 1 for details).

Figure 1 illustrates the set up of our simulations. Our models contain two crystalline layers—A and B—separated by semicoherent interfaces. Periodic boundary conditions are applied in all directions, so each model contains two A–B interfaces. Owing to their inherent internal structure, the interfaces create characteristic stress fields in the neighbouring crystalline layers. These stress fields interact with radiation-induced point defects, modifying their diffusion.

We calculate interface stress fields using an efficient, semi-analytical method developed previously^22^,^31^,^32^. This method accounts for the elastic anisotropy of solids A and B as well as for differences in elastic constants between them. It generates elastic field solutions consistent with a pre-specified interface crystallographic character (that is, misorientation and interface plane orientation^33^) and with vanishing far-field stresses. The method has been successfully applied to a variety of homo- and hetero-phase interfaces^22^,^31^,^32^ and has been validated through comparisons with atomistic simulations^18^.

Our modelling approach may be used on a wide range of semicoherent interfaces. However, for illustration, we will focus on two specific interfaces in the present work: a low-angle twist grain boundary (GB) on a (001) plane in Ag and a pure misfit (zero misorientation) heterophase interface between (001) planes of Ag and Cu. In our calculations, we use the lattice parameters and elastic constants for Ag and Cu listed in Table 1 (refs 34, 35).

**Figure 2:**
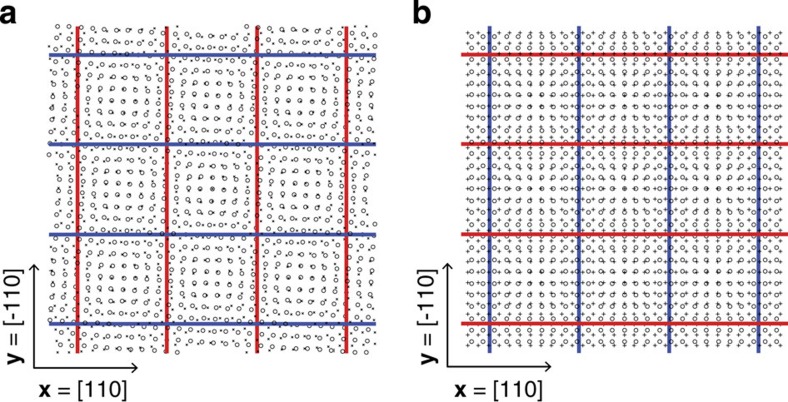
Planar semicoherent interfaces with identical misfit dislocation arrangements. (**a**) Ag twist GB with pure screw dislocations and (**b**) a Ag–Cu misfit interface with pure edge dislocations.

Figure 2a shows a plan view of the Ag twist GB, where the adjacent GB planes have been rotated by ±*θ*/2 (*θ*: twist angle). The boundary plane contains two sets of parallel, pure screw dislocations: one aligned with the **x**=[110] direction and the other with the 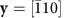 direction. For a relative twist angle of *θ*=7.5°, the spacing between dislocations within each set is ∼2.2 nm. Figure 2b shows the interface plane of the Ag–Cu pure misfit interface. Similar to the twist boundary in Fig. 2a, this interface also contains two sets of parallel dislocations aligned with the **x**=[110] and 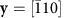 directions. Furthermore, the spacing between dislocations in the Ag–Cu interface is the same as in the twist boundary of Fig. 2a: ∼2.2 nm. However, unlike in the twist boundary, both sets of dislocations in the misfit interface are of pure edge type.

The two interfaces in Fig. 2 have identical dislocation arrangements, but different dislocation characters. Thus, they contain identical dislocation densities, but have differing stress fields. For instance, all normal stress components for the twist GB are zero throughout the entire bicrystal. This stress field is therefore purely deviatoric. By contrast, owing to symmetry, the shear stress *σ*_12_ is everywhere zero for the Ag–Cu interface, but all of its other stress components are in general non-zero. In particular, this interface generates significant hydrostatic stresses. These differences have important implications for interface-defect interactions and defect migration pathways.

We use the force dipole moment approximation to compute elastic interaction energies between point defects and interfaces, *E*^PD/int^ (refs 36, 37, 38):


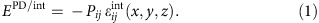


Here, 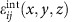 are the components of the previously calculated interface strain field. *P*_*ij*_ are the components of the elastic dipole tensor (the ‘**P**-tensor'), which describes the elastic fields generated by a point defect. *E*^PD/int^ values are used to compute stress-dependent energy barriers for defect migration at each location in the simulation cell (see Methods for details). A similar approach has been adopted in previous OKMC studies to describe point defect interactions with dislocations^39^,^40^.

We use density functional theory (DFT) to calculate **P**-tensors for two types of point defects in Ag and Cu (see Methods for details): vacancies and self-interstitials of lowest formation energy, namely 〈100〉-split dumbbells^41^. We obtain **P**-tensor values for these defects in their ground states as well as at their saddle point configurations during migration (found using the climbing image nudged elastic band method^42^). Starting from a simulation cell containing a perfect, stress-free crystal, we insert the point defect of interest in the desired location and relax the atom positions while keeping the simulation cell shape fixed. The point defect induces stresses, *σ*_*ij*_, in the simulation cell. They are related to the defect **P**-tensor through


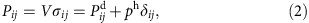


where *V* is the simulation cell volume. 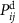 and *p*^h^ are the deviatoric and hydrostatic (isotropic) **P**-tensor components, respectively. The former is associated with a pure shear (no volume change), whereas the latter is related to isotropic tension (interstitials) or compression (vacancies), which leads to a volume change.

Table 2 lists the **P**-tensors used in the present study. All of them are expressed in the Nye frame, where the **X**, **Y** and **Z** axes are aligned with the [100], [010] and [001] Miller index directions, respectively. The form of the **P**-tensor reflects the symmetry of the corresponding defect. Thus, the **P**-tensor for a vacancy in its ground state is isotropic, whereas that of an interstitial is tetragonal. **P**-tensors for defect orientations other than those given in Table 2 may be calculated using coordinate system rotations. Our **P**-tensors for 〈100〉-split dumbbell self-interstitials and vacancies in Cu agree with experimental data^41^,^42^,^43^,^44^. Furthermore, the present calculations of relaxation volumes of a vacancy in Ag and Cu are in very good agreement with recent DFT predictions^45^.

**Figure 3:**
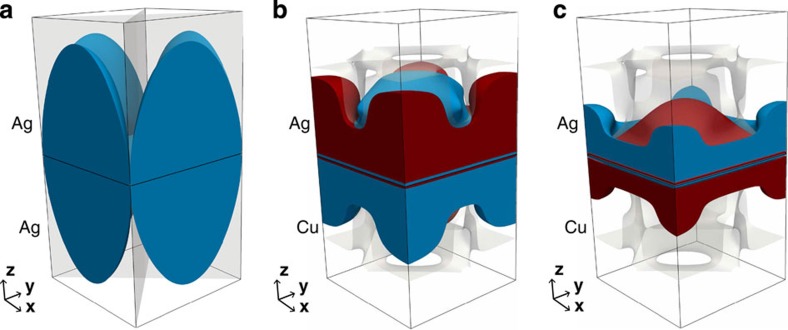
Elastic energy distributions around modelled interfaces. Elastic interaction energy between (**a**) an interstitial with the Ag twist GB (*E*^PD/int^<−0.002 eV in the blue isovolume), and between the Ag–Cu misfit interface with (**b**) an interstitial and (**c**) a vacancy (*E*^PD/int^<−0.06 eV in the blue isovolume; *E*^PD/int^>0.06 eV in the red; grey contours are locations with zero interaction energy).

Figure 3 shows the distribution of ground-state interstitial and vacancy interaction energies with the Ag twist GB and the Ag–Cu misfit interface. A 〈100〉-split dumbbell interstitial may take on three different orientations. Figure 3 uses the orientation with lowest *E*^PD/int^. For the Ag twist GB, interstitial interaction energies are negative at all locations, as shown in Fig. 3a. Thus, all interstitials in the vicinity of this GB experience a thermodynamic driving force to migrate towards the boundary. The interstitials, however, have nearly isotropic **P**-tensors (see Table 2), so their interaction energies with the Ag twist GB are very small. The interaction energy of vacancies with the Ag twist GB is everywhere zero because of the absence of hydrostatic stresses near this interface. However, the anisotropy of the vacancy saddle point configuration leads to non-zero interaction energies of migrating vacancies with the GB.

Interstitial interaction energies near the Ag–Cu misfit interface, shown in Fig. 3b, may be attractive or repulsive, depending on the location of the defect. Thus, interstitials in Ag are expected to migrate towards the centre of the dislocation pattern, whereas those in Cu are expected to migrate to dislocation cores. Figure 3c shows the interaction energy between vacancies and the Ag–Cu misfit interface. The spatial variation of this interaction energy is similar to that of the interstitials, but with opposite sign.

Our OKMC simulations assume a constant, uniform defect creation rate, *G*. Defects diffuse until they are absorbed by an interface (see Methods for details). Only individual interstitials or vacancies are tracked in our simulations: defect reactions, such as clustering or recombination, are not considered. After a certain simulation time, defect distributions reach a steady state, whereupon the defect concentration is computed as a function of position along the *z*-direction (normal to the interface plane) based on the time spent by each defect on a given atomic site.

### Effect of elastic interactions on interface sink strength

**Figure 4:**
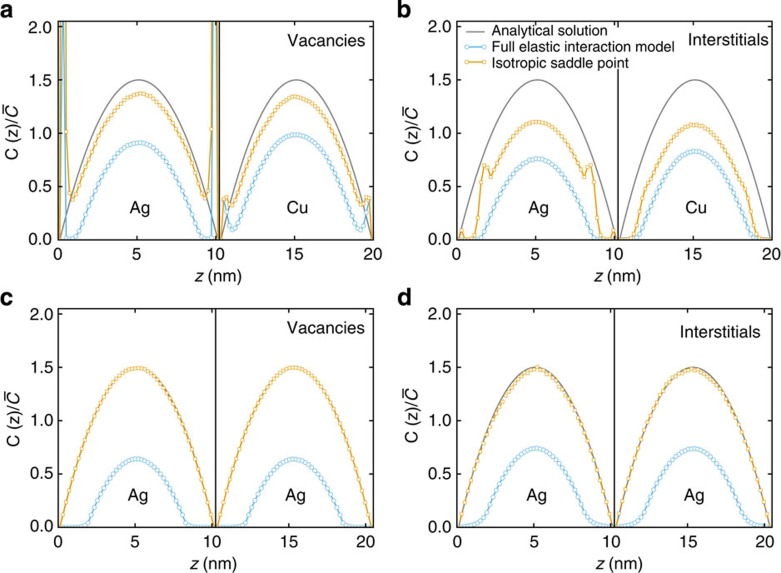
Steady-state point defect concentrations as a function of location normal to interface planes. The black vertical lines represent the interface planes, whereas the continuous grey lines denote the reference case with no elastic interactions, computed analytically (see Methods for details). OKMC results for both isotropic (orange) and anisotropic (blue) saddle point configurations are shown. (**a**) Vacancy and (**b**) interstitial profiles near Ag–Cu pure misfit interfaces. (**c**) Vacancy and (**d**) interstitial profiles near Ag twist GBs. Concentrations are normalized by the average concentration 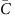 obtained when no elastic interactions are taken into account (see Methods for details).

Figure 4 shows steady-state vacancy and interstitial concentrations for the two types of interfaces described above for models with 10-nm-thick Ag and Cu layers. In the absence of elastic interactions between defects and interfaces, steady-state defect concentrations may be computed analytically (see Methods for details). We compare our simulation results with these analytical solutions.

Elastic interactions have a dramatic effect on defect concentration profiles. In all cases shown in Fig. 4 except vacancies near Ag–Cu interfaces, there are nearly no defects within ∼2-nm-wide zones adjacent to the interfaces. By contrast, without elastic interactions, defect concentrations are zero only at the interfaces themselves. Moreover, even though defect-interface elastic interaction energies are negligible beyond ∼2 nm, the zones depleted of defects near the interfaces have a pronounced effect on defect concentrations throughout the entire layer, markedly reducing the average defect concentration. For the simulations in Fig. 4, elastic interactions reduce defect concentrations by about a factor of two even in the middle of the layers. This effect is even more pronounced for thinner layers. For vacancies in Ag–Cu, local traps are responsible for the sharp increase in concentration near the interface.

Our simulations account for numerous aspects of defect-interface elastic interactions, such as defect anisotropy or differences in defect ground-state and saddle point properties. To discover which ones are primarily responsible for the defect concentrations shown in Fig. 4, we artificially ‘switch off' some of these characteristics and repeat our OKMC simulations to see whether doing so changes the steady-state defect concentrations. These calculations lead us to conclude that the anisotropy of the **P**-tensor in the saddle point configurations is primarily responsible for the reduced defect concentrations in Fig. 4a,b.

We ‘switch off' saddle point anisotropy by replacing the saddle point **P**-tensor with 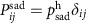, where *δ* is the Kronecker delta and 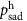 is one-third of the trace of the true saddle point **P**-tensor. This assumption is tantamount to modelling defects at saddle points as misfitting spherical inclusions in isotropic media. Concentration profiles obtained with this approximation are markedly different from the anisotropic case, as shown in Fig. 4. In the case of the Ag twist GB (Fig. 4c,d), isotropic saddle points yield the same defect concentrations as when there are no defect-interface interactions at all. Indeed, as the twist interface generates no hydrostatic strain field, only the deviatoric components of defect **P**-tensors may interact with these interfaces. Ground-state vacancies have zero deviatoric **P**-tensor components, so the interaction energy with the Ag twist GB vanishes, similar to ground-state interstitials with nearly isotropic **P**-tensors (Table 2). The same conclusions hold at saddle positions if saddle point anisotropy is ‘switched off', as described above. Elastic interactions then do not affect migration energies, explaining why defect concentrations are identical to the case without elastic interactions.

For the Ag–Cu interface, concentration profiles computed without saddle point anisotropy lie between the non-interacting and fully anisotropic cases, as shown in Fig. 4a,b. Vacancy concentrations are only marginally lower than the non-interacting case (Fig. 4a), demonstrating the overriding importance of saddle point anisotropy in their behaviour. Interstitial concentrations obtained without saddle anisotropy lie approximately mid-way between the fully anisotropic and non-interacting cases (Fig. 4b), demonstrating that saddle point anisotropy is at least as important to their behaviour as are *p*Δ*V* interactions, which are more commonly investigated.

**Figure 5:**
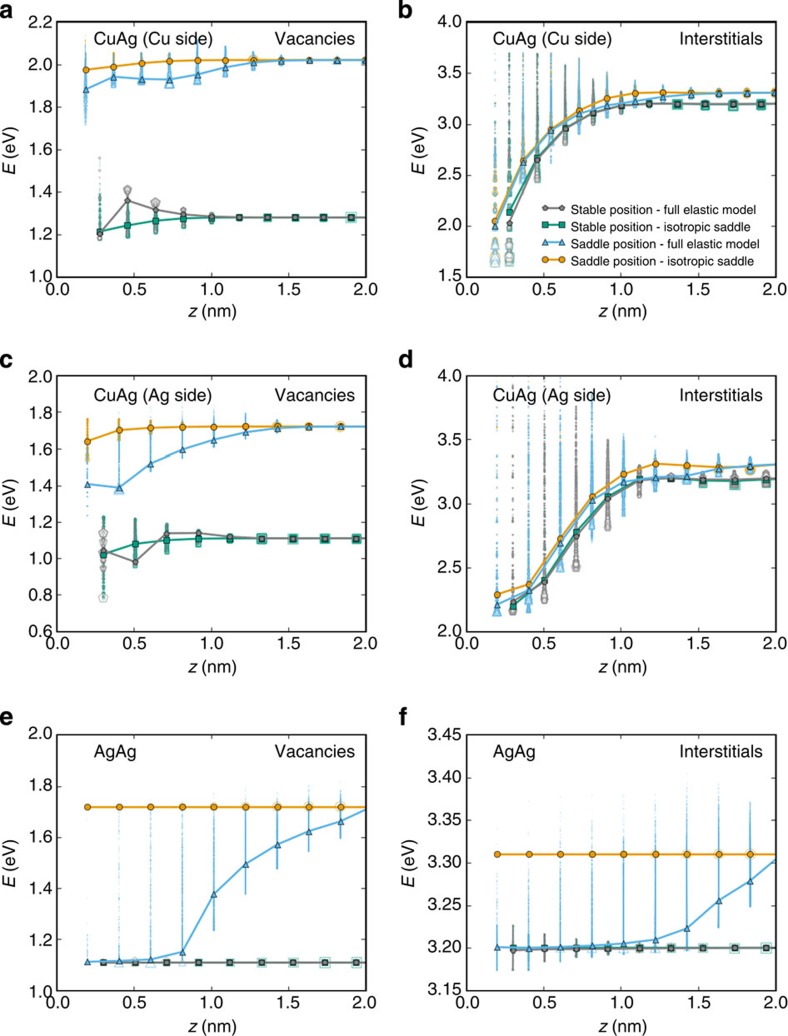
Effect of saddle point anisotropy on point defect energy landscapes. Mean stable (grey and green lines) and saddle (blue and orange) energy profiles as a function of distance to the interface for (**a**) vacancies and (**b**) interstitials on the Cu side and on the (**c**,**d**) Ag side of the Ag-Cu interface and for (**e**) vacancies and (**f**) interstitials of the Ag twist GB. Averages are computed over 10^4^ trajectories and the dispersion on the energy configurations explored by the defects is highlighted by the open symbols. The size of symbols is proportional to the times a defect passes through a given energy configuration. Orange (green) and blue (grey) lines correspond to simulations without saddle point anisotropy and with the fully anisotropic interaction model, respectively.

To investigate the effect of saddle point anisotropy on saddle point energy, Fig. 5 shows mean stable and saddle energy landscapes for point defects as a function of distance to the interfaces. Although the scatter in saddle point energies can be high at a given distance to interfaces (specially for interstitials near the Ag–Cu misfit interface), the mean saddle point energies (blue lines) are always lower than the mean saddle energies computed with the isotropic approximation (orange lines). For the Ag twist GB, the mean of the energies of saddle positions explored by vacancies and interstitials using the full elastic model decreases dramatically with decreasing distance to the GB. The case of vacancies near the Ag–Cu misfit interface illustrates the same trend, showing that the decrease in migration energy is due entirely to the saddle point configuration. In the case of interstitials near the Ag–Cu misfit interface, both migration energies and thermodynamic driving forces contribute to the enhancement of sink strength. In particular, a reduction of barriers is obtained when *z*<1.5 nm, thus giving rise to a non-random walk drift towards the Ag–Cu interface. The migration energy effect is especially pronounced in the case of vacancies and for interfaces without hydrostatic stresses.

This analysis therefore shows that, in addition to the classical thermodynamic driving force, reduced migration barriers contribute significantly to sink efficiency and that these reduced barriers are unequivocally due to saddle point anisotropy. The effect of interface elastic fields on defect migration energies is similar to that found in the vicinity of isolated dislocations^46^,^47^,^48^,^49^. However, elastodiffusion near interfaces—including semicoherent ones—is not reducible to elastodiffusion near dislocations because the stress fields of the former are in general composed of two contributions: one arising from infinite arrays of discrete dislocations and the other from coherency stresses. These contributions cancel perfectly in the far field, whereas in the near field the cancellation is imperfect^22^,^31^,^32^. It is only this imperfect cancellation in the near field that affects defect diffusion near interfaces.

**Figure 6:**
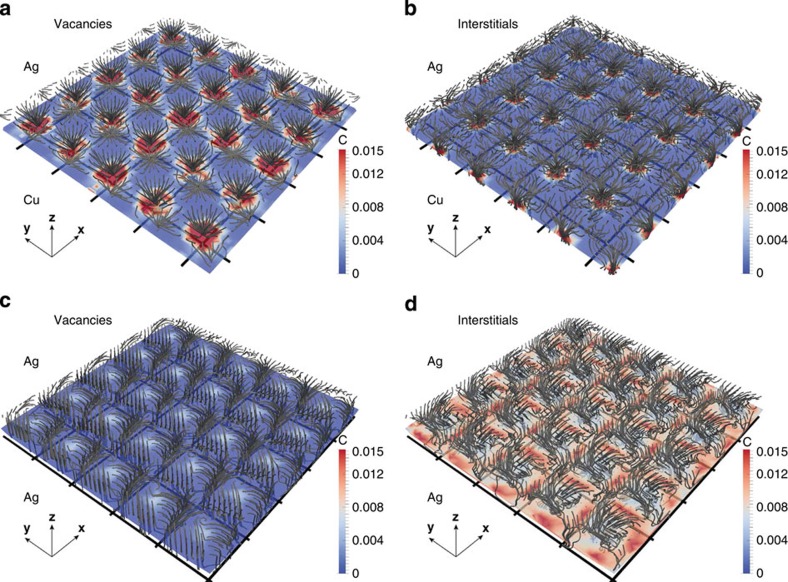
Preferential drift of point defects towards semicoherent interfaces. Migration paths and local concentrations of (**a**) vacancies and (**b**) interstitials on the Ag side of the Ag–Cu interface and of (**c**) vacancies and (**d**) interstitials in the Ag twist GB. Migration paths are shown as grey lines originating from 1 nm away from the interface. The square grid of black lines represents interface dislocations. Concentrations are plotted in a plane located two atomic distances away from the interface. The concentrations are normalized by 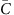: the average concentration when no interactions are considered. Any normalized concentration values higher than 0.015 are shown as equal to 0.015.

Figure 6 gives a more detailed view of defect concentrations at different locations in the Ag layer of the Ag–Cu interface and in the Ag twist GB. Close to these interfaces, concentrations vary as a function of location parallel to the interface plane, following the strain field pattern created by the interfaces. Indeed, the strain field creates preferential paths for defect migration, as shown by the grey trajectories in Fig. 6. These paths are in general different for interstitials and vacancies. For both the Ag–Cu interface and Ag twist GB, vacancies preferentially migrate to the dislocation lines, whereas interstitials are mostly absorbed between dislocations. This preferential, non-random walk drift of point defects to specific locations is responsible for the enhanced interface sink strengths.

Knowing the steady-state defect concentrations obtained by OKMC, we derive sink strengths for the two interfaces considered above. In the mean field rate theory formalism^50^, ‘sink strengths' quantify the ability of sinks, such as interfaces, to absorb defects. Within this formalism, the evolution equation for the average defect concentration, 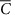, follows


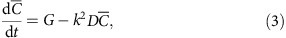


where *G* is the defect creation rate and *D* is bulk defect diffusivity. The second term on the right hand side is related to the loss of defects at sinks with associated sink strength, *k*^2^. At steady state, the sink strength may be computed from the average concentration:


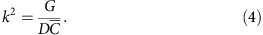


**Figure 7:**
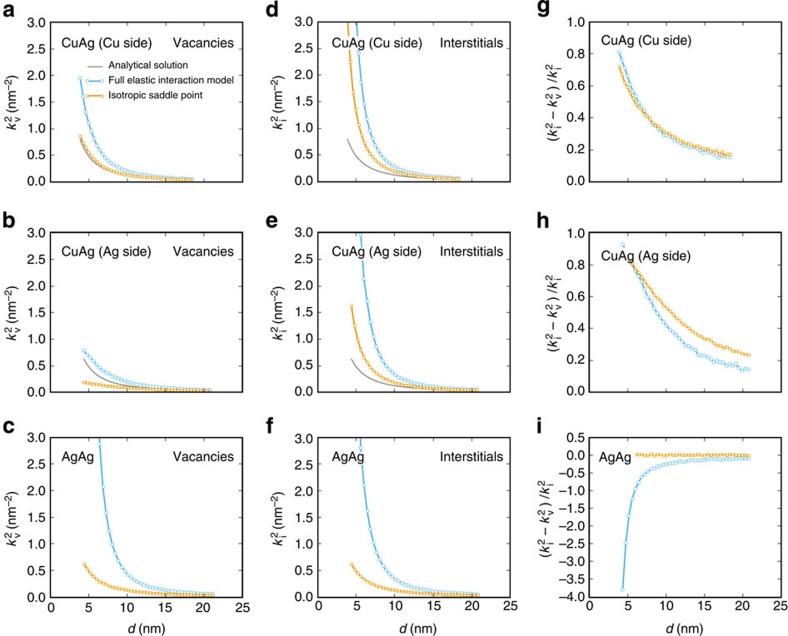
Enhancement in sink strength of semicoherent interfaces. Sink behaviours of Ag–Cu interfaces and Ag twist GBs for (**a**–**c**) vacancies 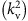 and (**d**–**f**) interstitials 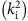 in a given layer (Ag or Cu), as a function of layer thickness, *d*. (**g**–**i**) Bias factors of Ag–Cu interface and Ag twist GB. The grey line corresponds to the analytical solution when no interaction is present (*k*^2^=12/*d*^2^). Orange and blue lines correspond to OKMC calculations without saddle point anisotropy and with the fully anisotropic interaction model, respectively.

Using the average of the concentration profile computed for defect removal at interfaces in the absence of elastic interactions (see Methods for details), the interface sink strength is analytically found to be *k*^2^=12/*d*^2^ (ref. 51). When interactions between interfaces and defects are present, we numerically determine the sink strength through equation (4), by using the average steady-state concentration obtained by OKMC simulations and the diffusion coefficient without elastic interactions. The resulting vacancy and interstitial sink strengths for both interfaces are shown in Fig. 7a–f as a function of layer thickness.

In all cases, the sink strength increases significantly when elastic interactions are taken into account. This effect is especially pronounced for thinner layers, as defects undergo elastic interactions with interfaces over a larger fraction of the layer. It is particularly strong for interstitials, whatever the interface type, and for vacancies for the twist interface. These results also confirm the importance of saddle point anisotropy: by comparing with OKMC simulations that use isotropic saddle-point **P**-tensors, we see that it yields order-of-magnitude increases in sink strength, in some cases.

Another quantity of interest for radiation response is the bias factor, *B*, which expresses the propensity of a given sink to absorb more interstitials than vacancies. It is defined as


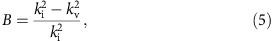


where 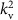 and 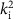 are the sink strengths for vacancies and interstitials, respectively. For example, small interstitial clusters and dislocations exhibit positive bias factors (typically between 0.01 and 0.3 (refs 52, 53)) and thus absorb more interstitials than vacancies. The preferential absorption of interstitials by biased sinks leads to an excess of remaining vacancies, which cluster and eventually aggregate into voids^52^,^54^.

Bias factors for the semicoherent interfaces we studied are shown in Fig. 7g–i. Values larger than 0.2 are obtained for the fully anisotropic interaction model in the case of the Ag–Cu interface. Such interfaces would compete for interstitials with dislocations. The presence of two sinks of differing bias magnitude has been given as a possible cause for void swelling suppression in ferritic steels^55^. Interestingly, for the Ag twist GB, the bias factor is negative, meaning that these interfaces tend to absorb more vacancies than interstitials. Similar observations have been made in ref. 56, where the bias factor for single screw dislocations is negative when using anisotropic elasticity theory and zero in the isotropic approximation. Such GBs may therefore deplete excess vacancy concentrations sufficiently to inhibit void nucleation.

## Discussion

Our work demonstrates that elastic interactions between radiation-induced point defects and semicoherent interfaces lead to significant increases in interface sink strength, compared with the case with no defect-interface interactions. These conclusions are consistent with other recent simulations, which show that elastic interactions also have a significant influence on defect-dislocation interactions^39^,^40^,^56^ and on mutual interactions between radiation-induced dislocation loops^57^,^58^,^59^.

Our simulations identify non-hydrostatic interactions of point defects in saddle point configurations as the main cause of enhanced interface sink strengths. How might one incorporate such interactions into continuum-level diffusion models? Compared with diffusion in the absence of stress fields, the effect of local variations in defect ground-state and saddle point energies is twofold^44^,^60^: the diffusivity gains a spatial dependence and the continuum diffusion equation gains a drift term. These modifications are sufficient to model defects that undergo purely hydrostatic, *p*Δ*V* interactions with elastic fields. When non-hydrostatic interactions are considered, local diffusivities are, in general, no longer isotropic and the scalar diffusivity must be replaced by a diffusivity tensor. The effect of saddle point anisotropy on vacancy diffusion may be modelled this way.

Modelling the effect of saddle point anisotropy on the diffusion of 〈100〉-split dumbbell self-interstitials furthermore requires tracking the orientation of the interstitials. Therefore, three separate interstitials concentrations (corresponding to the three different dumbbell orientations) must be defined^44^. As these interstitials re-orient during each migration step (see Methods for details), the evolution of their concentrations is described by three coupled diffusion equations, each with a different location-dependent diffusivity tensor. It may also be necessary to account for thermally activated interstitial re-orientation without migration^61^. Posing and solving a continuum model for such a complex process is likely to remain beyond the scope of most continuum modelling studies in the near future. Thus, OKMC simulations are likely to remain the workhorse method for investigating the effect of elastic interactions on point defect diffusion, especially of self-interstitials.

Our simulations show that the Ag twist GB and the Ag–Cu interface have markedly different sink strengths, even though both have identical dislocation densities. These differences arise from unlike detailed strain distributions, which are due to the different dislocation characters in these interfaces and the unlike coherency strains in the reference states of each interface. The orientations of the adjacent crystals in the twist GB are related through a pure rotation, so the GB has no coherency strains. By contrast, in the Ag–Cu pure misfit interface, unequal stresses of opposite sign are needed to impose coherency^22^,^31^,^32^.

The sensitivity of interface sink strength to interface elastic fields opens up new opportunities for materials design. For instance, ‘super-sink' interfaces with maximal sink strengths may be created by optimizing interface elastic fields. Interface elastic fields may also be designed to yield a desired bias factor. The dependence of interface elastic fields on interface crystallography and the physical properties of the adjoining crystals are well-established^18^,^22^. Strategies for scanning the interface design space and locating optimal interface crystallographies and compositions have also been developed^18^,^62^.

An advantage of the OKMC-based simulation method presented here is that it may be systematically improved to enhance its accuracy. For example, our simulations did not take into account the interactions of defects with each other. One extension of the model would therefore be to include defect reactions, such as vacancy-interstitial recombination or clustering of like defects^63^. Another simplifying assumption of the current simulations is that they consider only the first-order term in the interaction energy between interfaces and defects^44^. An improved model might account for other interactions, such as higher order moments of the multipole expansion^37^ or heterogeneity interactions^64^. The internal structure of interfaces may change because of loading of point defects^15^. Our computational method may be further improved by modelling this structure evolution, for example, following the approach of Uberuaga *et al.*^65^. Finally, all of our simulations assumed perfect trapping of defects that arrive at an interface (see Methods for details), which corresponds to an interface sink efficiency of unity^66^. This assumption may be relaxed to model the sink efficiency of interfaces that are not perfect defect sinks.

## Methods

### Interface elastic field calculation

We compute the complete elastic strain fields *ɛ*^int^ of interface dislocation arrays using the method described in ref. 22. The geometry of the interface dislocation pattern is found by solving the quantized Frank–Bilby equation^33^. Interface dislocations are viewed as Volterra dislocations that have been inserted into a single crystal, coherent reference interface. The complete interface strain is the superposition of the coherency strain *ɛ*^coh^ in the reference interface and the strain field of the interface dislocations *ɛ*^int^:


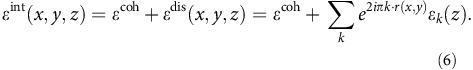


*ɛ*^dis^ is represented using a Fourier expansion on the right hand side of the expression above. The dislocation strain field must satisfy mechanical equilibrium in both of the adjacent crystals:


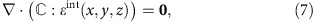


where 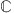 the fourth-order anisotropic elasticity tensor. Substituting equation (6) into equation (7), the dislocation strain field is obtained by solving the sextic eigenvalue problem developed by Stroh^67^ with specific boundary conditions dedicated to interface dislocations:

- No net far-field strains.
- Consistency of far-field rotations with the prescribed interface misorientation.
- No net tractions along the interface.
- Interface displacement discontinuity matches the disregistry of the desired dislocation pattern.

### Elastic dipole tensor calculation

Defect **P**-tensors are calculated using VASP (the Vienna Ab initio Simulation Package^68^), a plane wave-based, first principles DFT code. A face-centred cubic supercell containing 256±1 atoms (+1 and −1 for interstitial and vacancy, respectively) is used. We also performed LAMMPS (Large-scale Atomic/Molecular Massively Parallel Simulator^69^) classical potential simulations using embedded atom method potentials for Ag (ref. 70) and Cu (ref. 71) to study the convergence of the elastic dipole tensors up to supercell sizes of 2,048 atoms. We find that the discrepancy in **P**-tensor components between the 256-atom supercell and that of 2,048-atom supercell is lower than 4%. This supercell size ensures the convergence of defect formation energies to within few meV, as detailed in the Supplementary Note and Supplementary Figs 1 and 2. We therefore view the 256-atom DFT simulations as well converged with respect to model size.

A 3 × 3 × 3 shifted Monkhorst–Pack *k*-point grid mesh, a Hermite–Gaussian broadening of 0.25 eV (ref. 72) and a plane wave cutoff energy of 400 eV are used. The change of the elastic dipole tensors is less than 0.5% compared with tighter settings. We use the Perdew–Burke–Ernzerhof^73^ exchange-correlation functional within the projector-augmented-wave approach^74^. The structures are internally relaxed with a force convergence criterion of 10^−3^ eV Å^−1^. The climbing image-nudged elastic band method^42^ is employed to find the saddle points for defect migration.

**Figure 8:**
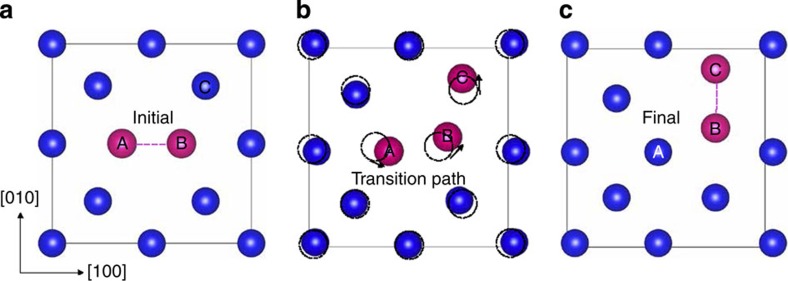
The migration mechanism of a 〈100〉-split dumbbell interstitial in a face-centred cubic crystal. (**a**) Initial dumbbell configuration, (**b**) saddle point configuration and (**c**) final dumbbell configuration. Initial atom positions are shown by dashed circles in **b**.

In the migration of a vacancy, one of the atoms directly neighbouring the vacancy travels to the vacant site, leaving behind a new vacancy. The more complex migration mechanism of a 〈100〉-split dumbbell interstitial is shown in Fig. 8. Here, one atom (B) in the initial dumbbell configuration (A−B, Fig. 8a) migrates to a neighbouring lattice site (C), forming a new 〈100〉 dumbbell (B−C, Fig. 8c). Thus, three atoms (A, B and C) are involved in this migration mechanism. The initial and final dumbbells, as well as the transition path, are confined to the same lattice plane. The initial and final dumbbell orientations are orthogonal to each other.

### OKMC algorithm

We model defect diffusion using an OKMC code with a residence time algorithm to advance the simulation clock^27^,^28^. At time *t*, the time step is chosen according to Δ*t*=−(ln *r*_1_)/*w*_tot_, where *r*_1_ is a random number with *r*_1_∈]0,1] and *w*_tot_ is the sum of frequencies of all events 
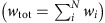
 that may occur at *t*. The chosen event *j* is such that 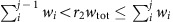, where *r*_2_ is another random number with *r*_2_∈]0,1].

Three kinds of events are considered in the simulations: the jump of a point defect from one stable point to a neighbouring one, the absorption of a defect by an interface and the creation of a new point defect through irradiation. Jump frequencies are given by *w*_*i*_=*ν*exp(−Δ*E*_*i*_/(*kT*)), where ν is an attempt frequency and 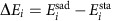 is the energy difference between the saddle position and the initial stable position of the jump considered. The stable point energy is


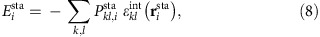


whereas the saddle point energy is


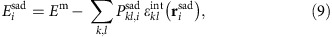


with *E*^m^ the migration energy in the absence of elastic interactions.

Here, **P**^sta^ and **P**^sad^ are the defect **P**-tensors in the ground-state and saddle point configurations, respectively. For simplicity, the position of the saddle point 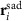 is taken mid-way between the two stable points explored by the jump^40^.

The defect is considered to have been absorbed by an interface if it reaches the nearest atomic row to the interface. It is then simply removed from the simulation. This absorption condition is used to obtain a first estimate of sink strength, without taking into account the diffusion of point defects along interfaces or their possible reemission. The irradiation rate is fixed at the beginning of each simulation to keep the average number of point defects equal to 200 in the material where the measurements are performed, if no elastic interactions are considered. The actual number of point defects in the system, averaged over the simulation time when steady state is reached, constitutes the basis for our sink strength calculation.

The concentration of defects is recorded every 10^4^ iterations, after the concentration has become stationary. At the end of the simulation, an estimate of 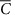 is computed by averaging over the values *C*_*j*_ (*j*=1,…,*n*):


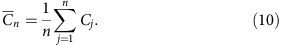


The final time is adjusted to obtain sufficient accuracy on 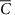 and thus on *k*^2^. For this purpose, the estimation of the error on the concentration is given by the standard error of the mean value, that is,


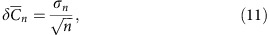


where


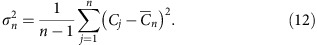


The final time for each system is chosen so that the relative error on 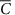 and *k*^2^ is less than 0.5%.

### Analytical solution in the absence of elastic interactions

When there are no defect–interface interactions, the steady-state concentration in a flat crystalline layer may be found analytically. We consider a layer of thickness *d* with interfaces at *z*=−*d*/2 and *z*=*d*/2, where zero concentration Dirichlet conditions are imposed. Defects are created by radiation at a constant rate, *G* (per atomic site and per second) and diffuse with a diffusion coefficient *D*. Solving the steady-state diffusion equation leads to


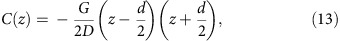


from which the average concentration 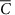 per atomic site is readily deduced:


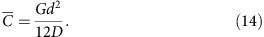


Taking into account admissible jumps for a 〈100〉-split dumbbell interstitial in a face-centred cubic lattice, this interstitial diffusion coefficient is


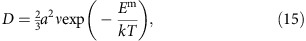


whereas in the case of vacancies it is


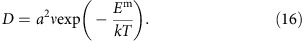


## Additional information

**How to cite this article:** Vattré, A. *et al.* Non-random walk diffusion enhances the sink strength of semicoherent interfaces. *Nat. Commun.* 7:10424 doi: 10.1038/ncomms10424 (2016).

## Supplementary Material

Supplementary Information Supplementary Figures 1- 2, Supplementary Note 1 and Supplementary References.

## Figures and Tables

**Table 1:** Materials properties for Silver (Ag) and Copper (Cu).

**Element**	**Cubic lattice parameter (Å)**	**Elastic constants (GPa)**		
		***c***_**11**_	***c***_**12**_	***c***_**44**_
Ag	4.086	124.0	93.4	46.1
Cu	3.615	168.4	121.4	75.5

The lattice parameters and elastic constants of Ag and Cu are used to compute anisotropic elastic fields generated by the semicoherent interfaces with misfit dislocations.

**Table 2:** Elastic dipole tensors of point defects from first principles.

**Element**	**Interstitial**		**Vacancy**	
	**Ground state**	**Saddle point**	**Ground state**	**Saddle point**
Ag	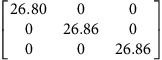	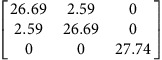	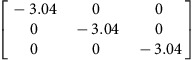	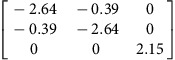
Cu	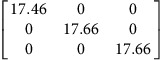	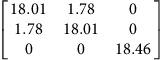	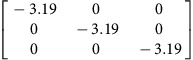	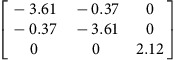

**P**-tensors (in eV) for a 〈100〉-split dumbbell self-interstitial and a vacancy in Ag and Cu at both the ground-state and saddle point configurations. The ground-state interstitial is oriented in the [100] direction. Its saddle point configuration is for a [100]-to-[010] migration path (see Methods for details). The vacancy saddle point is for migration along the [110] direction.
