# Supplementary material for: Non-random walk diffusion enhances the sink strength of semicoherent interfaces
Source: Nat Commun. 2016 Jan 29;7:10424. doi: 10.1038/ncomms10424 (PMC4740113; doi:10.1038/ncomms10424)
Supplement: Supplementary Information — Supplementary Figures 1- 2, Supplementary Note 1 and Supplementary References. [file ncomms10424-s1.pdf]

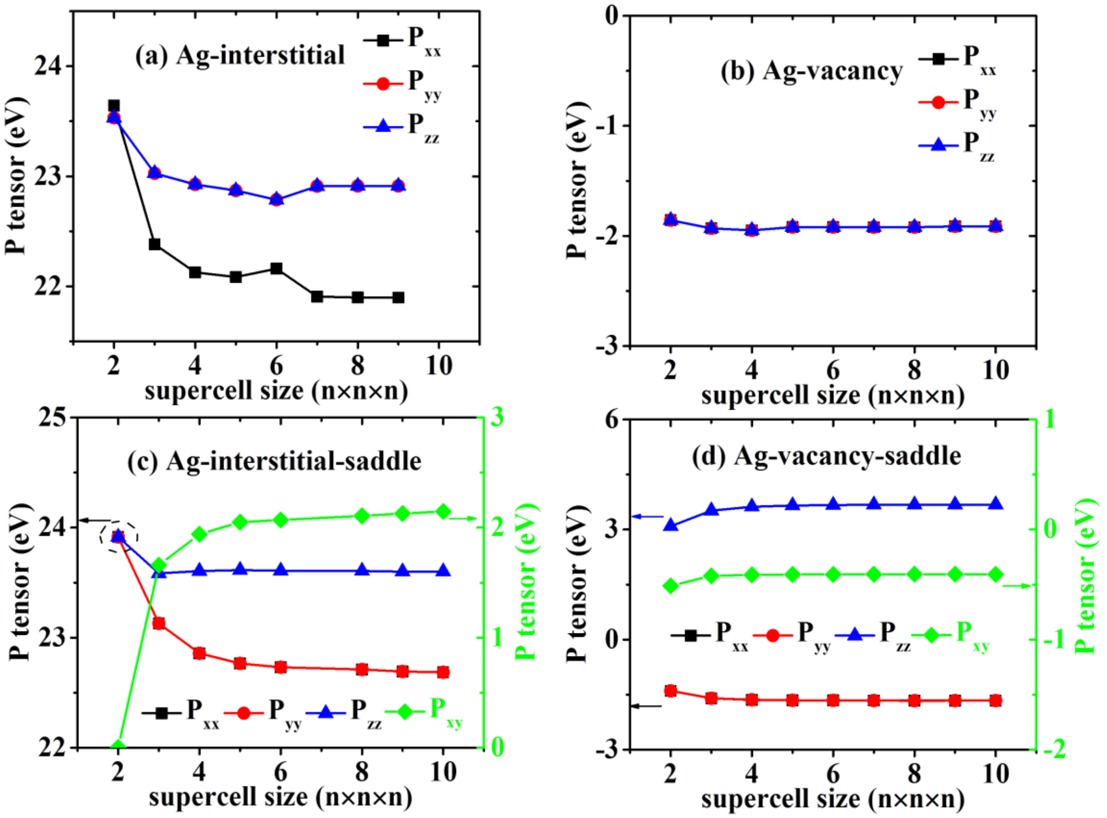

Supplementary Figure 1

Convergence of **P**-tensors with respect to the supercell size for Ag. (a) [100]-split dumbbell self-interstitial at the ground state, (b) vacancy at the ground state, (c) [100]-split dumbbell self-interstitial at the saddle point configuration along the [100]-to-[010] migration path, (d) vacancy at the saddle point configuration for migration along the [110] direction.

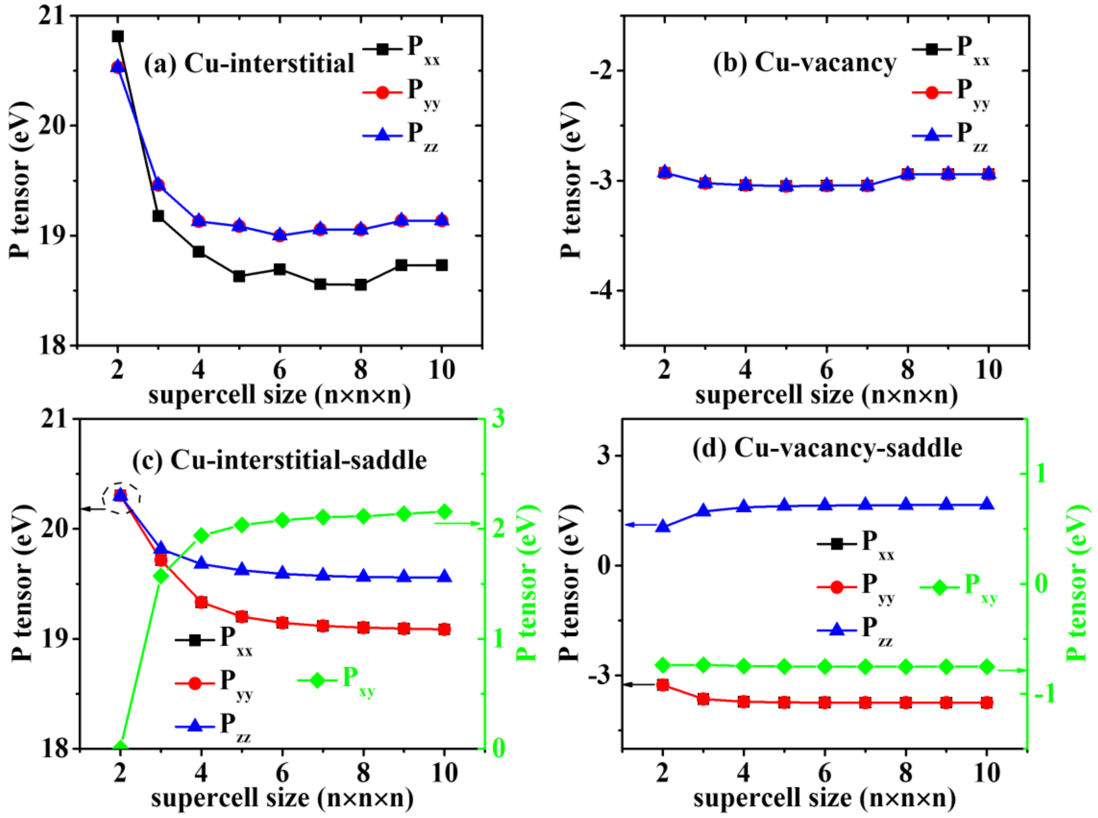

Supplementary Figure 2

Convergence of **P**-tensors with respect to the supercell size for Cu. (a) [100]-split dumbbell self-interstitial at the ground state, (b) vacancy at the ground state, (c) [100]-split dumbbell self-interstitial at the saddle point configuration along the [100]-to-[010] migration path, (d) vacancy at the saddle point configuration for migration along the [110] direction.

Supplementary Note 1

Convergence of P-tensors with respect to supercell size

P-tensors are extracted using atomistic simulations in supercells under periodic boundary conditions. We carry out convergence tests of these **P**-tensor calculations with respect to the size of the supercell using LAMMPS [1] classical potential simulations with embedded atom method (EAM) [2] potentials for Ag [3] and Cu [4]. In all tests, the lattice constants are fixed at their lowest energy values for both potentials, namely 4.090 Å for Ag and 3.615 Å for Cu. Supercells of Ag and Cu in the face-centered cubic structure ranging from 2×2×2 (32 atoms) cubic cells to 10×10×10 (4000 atoms) cubic cells are studied. The dependence of **P**-tensor values on the supercell size is shown in Supplementary Figure 1 and Supplementary Figure 2 for Ag and Cu, respectively. In all cases considered, the 256-atom (4×4×4) simulations are well converged as the discrepancies in **P**-tensor components between the 256-atom supercell and that of 4000-atom supercell are lower than 4%.

Supplementary References

[1]. Plimpton S. Fast parallel algorithms for short-range molecular-dynamics. J. Comput. Phys. 117, 1-19 (1995).

[2]. Daw MS, Baskes MI. Embedded-atom method - derivation and application to impurities, surfaces, and other defects in metals. *Phys. Rev. B* **29**, 6443-6453 (1984).

[3]. Foiles SM, Baskes MI, Daw MS. Embedded-atom-method functions for the FCC metals Cu, Ag, Au, Ni, Pd, Pt, and their alloys. *Phys. Rev. B* **33**, 7983-7991 (1986).

[4]. Mishin Y, Mehl MJ, Papaconstantopoulos DA, Voter AF, Kress JD. Structural stability and lattice defects in copper: *Ab initio*, tight-binding, and embedded-atom calculations. *Phys. Rev. B* (Condensed Matter and Materials Physics) **63**, 224106-224116 (2001).
